# Growth-Associated Ecological Reorganization of the Gut Microbiota in *Larimichthys crocea*: Shifts in Microbial Persistence and Niche Breadth

**DOI:** 10.3390/biology15201807

**Published:** 2026-10-10

**Authors:** Fang Liu, Ding Li, Peixin Wu, Zuhan Chen, Fangyu Yuan, Kunhuang Han, Weiqing Huang

**Affiliations:** 1 College of Marine Sciences, Ningde Normal University, Ningde 352100, China; lf_ndsy@163.com (F.L.); wupeixinxz@163.com (P.W.); ch20060401@outlook.com (Z.C.); 2 Fujian Provincial University Engineering Research Center for Deep Processing of Mindong Aquatic Products, Ningde 352100, China; 3 Haixi Public Platform for Marine Characteristic Germplasm Resources and Biological Products Development, Ningde 352100, China; 4 School of Medicine, Fuzhou University, Fuzhou 350108, China; t25116@fzu.edu.cn

**Keywords:** *Larimichthys crocea*, gut microbiota, growth stage, microbial succession, persistent taxa, niche breadth

## Abstract

Gut microbial communities undergo continuous reorganization during fish development, but changes in microbial persistence and ecological niche strategies across post-juvenile growth remain insufficiently understood. Here, we characterized the gut bacterial communities of cultured *Larimichthys crocea* across three successive body-size classes: EGC (5–8 g), MGC (39–46 g), and LGC (160–162 g). The three stages were successively sampled from the same cultured population maintained in the same culture pond. At each stage, 15 fish were sampled, and intestinal samples from three individuals were pooled to generate one pooled intestinal sample, yielding five pooled samples per stage. Fish received the same feed formulation throughout cultivation, while feeding quantity was adjusted according to growth. Rarefaction analysis indicated adequate sequencing coverage, and ASV-level community structure differed significantly among growth stages (ANOSIM, R = 0.4698, *p* = 0.001). *Pseudomonadota* remained the dominant phylum throughout growth, whereas pronounced restructuring emerged at finer taxonomic resolutions. EGC communities showed a comparatively consistent composition dominated by *Methylobacterium*, while MGC and particularly LGC exhibited substantial redistribution among dominant bacterial lineages. Stage-specific membership also changed with growth: unique genera increased from 36 in EGC to 48 in MGC and 50 in LGC, whereas stage-specific ASVs showed a nonlinear pattern and peaked in MGC (118, 173, and 124 unique ASVs in EGC, MGC, and LGC, respectively); 88 ASVs were shared across all three stages. Persistent taxa remained numerically important, accounting for 36.34%, 31.59%, and 36.95% of detected taxa in EGC, MGC, and LGC, respectively, but their contribution to total community abundance declined from 90.30% to 75.29% and 68.06%. Conversely, intermittent taxa increased in abundance contribution from 6.80% to 14.85% and 29.17%. Niche-breadth analysis further showed that specialists represented 74.38% of classified taxa and generalists 19.26%. Although generalists remained quantitatively dominant, their contribution declined from EGC to LGC, whereas specialist contribution increased. Growth-associated taxa further showed contrasting trajectories, particularly a progressive decline in *Methylobacterium* and increase in Aquabacterium. Collectively, these findings reveal pronounced ecological reorganization of the gut microbiota across successive size- and age-associated sampling stages of *L. crocea*. Because body size and age covaried with chronological sampling time in a single culture pond, these patterns should be interpreted as growth-associated temporal restructuring rather than as effects attributable solely to host growth.

## Simple Summary

The intestinal microbiota of *Larimichthys crocea* changes as fish grow, but these changes do not necessarily follow a simple progression toward a more stable community. In this study, fish from the same cultured population were maintained in the same culture pond and sampled successively at three body-size stages. The dominant bacterial phylum remained broadly conserved, whereas substantial changes occurred at finer taxonomic levels. Persistent microorganisms remained common across the growth sequence but contributed progressively less to total community abundance, while intermittently occurring microorganisms became more quantitatively important. In parallel, microorganisms classified as ecological specialists represented most detected taxa and contributed increasingly to later-stage communities, although generalists remained dominant in overall abundance. These findings show that gut microbiome development in *L. crocea* involves redistribution of microbial dominance and ecological strategies rather than simple microbial stabilization.

## 1. Introduction

The gastrointestinal tract of fish supports complex microbial communities that form an integral component of the host-associated ecosystem. These microorganisms are associated with nutrient transformation, intestinal development, immune regulation, colonization resistance, and host responses to environmental and dietary variation [1,2]. Unlike terrestrial vertebrates, fish continuously interact with microorganisms originating from rearing water, feed, sediment, and other components of the aquatic environment. Nevertheless, intestinal communities are not simply passive reflections of environmental microbial pools, because host physiology and gut habitat conditions can selectively retain particular bacterial populations [3,4,5]. Comparative analyses across fish species have consequently shown that host ecology, diet, developmental stage, and environmental conditions jointly contribute to gut microbial community structure [3,4].

Host growth represents an especially important dimension of fish microbiome variation. Ontogeny is accompanied by changes in intestinal morphology, digestive capacity, immune development, food intake, nutrient demand, and gut physicochemical conditions, all of which can alter the ecological opportunities available to microorganisms [1,5]. Pronounced developmental succession has been documented in *Danio rerio*, *Ictalurus punctatus*, *Salmo salar*, and *Gadus morhua* [6,7,8,9]. *Danio rerio* intestinal communities change substantially across host development, while *Ictalurus punctatus* exhibit strong temporal turnover and distinct later-stage microbial configurations [6,7]. *Salmo salar* likewise show stage-specific bacterial signatures during development, and studies of cod larvae demonstrate that host developmental processes can contribute substantially to microbial community restructuring [8,9].

Developmental changes in fish gut microbiota, however, are not necessarily unidirectional. Fish communities do not universally progress from a heterogeneous early assemblage toward a uniformly stable adult microbiota. Ecological assembly processes themselves may change during host development, and the relative importance of environmental filtering can vary substantially with developmental stage [10]. Early-life studies of *Trichinotus Blochii* similarly demonstrate that transient and recurrent microbial components can shift substantially during ontogeny [11]. These observations highlight the importance of evaluating microbial development in terms of ecological organization, rather than assuming that host growth necessarily produces progressively greater microbiome stability. Core microbiome studies in *Danio rerio* and other fishes have demonstrated that recurrent bacterial populations can persist across hosts, environmental conditions, diets, or time [12,13]. Microbial niche breadth provides a complementary ecological dimension. Generalists are capable of occupying broader ecological contexts, whereas specialists are associated with narrower realized niches. Studies across large microbial datasets have shown that generalists and specialists can exhibit distinct distributional, evolutionary, and community-level characteristics [14,15]. The ecological distinction is relevant to host-associated microbiomes because growth-related changes in intestinal conditions could alter the relative contribution of broadly distributed versus more niche-restricted microorganisms. Persistence and niche breadth represent distinct ecological dimensions: a persistent taxon is not necessarily a generalist, and a specialist can occur consistently within a specific host environment.

*L. crocea* is an important marine aquaculture species in China, and its intestinal microbiota has received increasing attention. Previous studies have demonstrated substantial age-related changes in its intestinal bacterial composition [16], while recent integrated transcriptome–microbiome analyses revealed stage-specific microbial succession during digestive-system development [17]. Dietary interventions, including fucoidan supplementation, fermented soybean meal, alternative animal-protein ingredients, probiotics, and antimicrobial peptides, can also modify the intestinal microbiota of *L. crocea* [18,19,20,21,22]. Recent work further indicates associations between intestinal microbiota and feed efficiency, growth variation, rearing environment, and disease status in this species [23,24,25,26]. Despite these advances, relatively little is known about how microbial occupancy and niche strategies change across later growth stages of *L. crocea* when feed formulation remains constant. Understanding this ecological dimension is important because compositional turnover alone cannot reveal whether growth changes the dominance of persistent community members or alters the balance between microbial generalists and specialists.

In the present study, gut bacterial communities were examined in *L. crocea* across three successive body-size classes: EGC (5–8 g), MGC (39–46 g), and LGC (160–162 g). The three stages were successively sampled from the same cultured population maintained in the same culture pond. Fish received the same feed formulation throughout cultivation, while feeding quantity was adjusted according to host growth. Using 16S rRNA gene amplicon sequencing, we evaluated community structure, taxonomic composition, stage-specific microbial membership, occurrence-based persistence categories, microbial niche breadth, and growth-associated bacterial taxa.

## 2. Materials and Methods

### 2.1. Experimental Design and Sample Collection

Cultured *L. crocea* used in this study originated from the same cultured population and were maintained in a single culture pond throughout the study period. The first sampling point was defined as month 0 of the present study, when the fish were approximately 3 months old and weighed 5–8 g; these fish were designated as EGC. The second sampling was conducted after an additional 2 months of culture, when the fish were approximately 5 months old and weighed 39–46 g; these fish were designated as MGC. The third sampling was conducted after 5 months of culture from the initial sampling point, when the fish were approximately 8 months old and weighed 160–162 g; these fish were designated as LGC. Thus, the three groups represented successive size- and age-associated sampling stages from the same cultured population rather than independent experimental treatments.

Fish were continuously fed the same formulated diet throughout the study period, and feed composition remained unchanged among the three sampling stages. Feeding quantity was adjusted according to fish size and feeding requirements. Concurrent records of water temperature, salinity, dissolved oxygen, pH, ammonia, and nitrite were not available for the three sampling periods; therefore, possible temporal variation in the rearing environment could not be quantitatively evaluated in the present study.

At each sampling stage, 15 apparently healthy fish were randomly collected from the culture pond. Before tissue collection, fish were anesthetized with tricaine methanesulfonate (MS-222, Syndel, Ferndale, MI, USA; 200 mg L^−1^) until complete loss of opercular movement and response to external stimuli, followed by euthanasia. Fish were fasted for 12 h before sampling to reduce the influence of recently ingested feed and intestinal digesta. The 15 fish were divided into five subsets, with three fish in each subset. After euthanasia, the intestine was aseptically excised from each fish. Intestinal contents were removed, and the intestinal tissue was gently rinsed with sterile water to minimize residual luminal material. Intestinal tissues from three fish within each subset were then pooled to generate one pooled intestinal sample. Consequently, five pooled intestinal samples were obtained at each sampling stage, resulting in 15 samples in total. The five pooled samples represented biological subsamples obtained from fish within the same culture pond and should not be interpreted as independent pond-level replicates. In addition, because only five pooled samples were available per sampling stage, the design provides limited statistical power for detecting between-stage differences and does not allow assessment of microbiome variation among individual fish. Samples were designated EGC1–EGC5, MGC1–MGC5, and LGC1–LGC5. The pooled intestinal tissues were immediately transferred into sterile cryovials, rapidly frozen, and stored at −80 °C until DNA extraction.

Body length, sex, sex ratio, condition factor, and gonadal maturation status were not systematically recorded at the time of sampling and therefore could not be retrospectively included in the present analysis.

### 2.2. Total Genomic DNA Extraction and Quality Assessment

Microbial genomic DNA was extracted from the pooled, rinsed intestinal tissue samples using the DNeasy PowerSoil Pro Kit (QIAGEN GmbH, Hilden, Germany) according to the manufacturer’s instructions. Because intestinal contents were removed and the intestinal tissues were rinsed before pooling, the resulting microbial profiles primarily represented microorganisms associated with the intestinal tissue and mucosal surface rather than the luminal digesta microbiota. Before DNA extraction, pooled intestinal tissues were aseptically homogenized to obtain representative microbial DNA.

The concentration of extracted DNA was measured using a Qubit 3.0 Fluorometer (Life Technologies, Thermo Fisher Scientific, Waltham, MA, USA) with the Equalbit 1× dsDNA HS Assay Kit (Vazyme, Nanjing, China). DNA integrity and purity were further evaluated using 1.0% agarose gel electrophoresis and a NanoDrop ND-2000 spectrophotometer (Thermo Scientific, Waltham, MA, USA). DNA samples meeting the quality requirements were subsequently used for 16S rRNA gene amplification.

### 2.3. Amplification of the 16S rRNA Gene

The V4 hypervariable region of the prokaryotic 16S rRNA gene was amplified using the primer pair 515FmodF (5′-GTGYCAGCMGCCGCGGTAA-3′) and 806R (5′-GGATACHVGGGTWTCTAAT-3′). PCR amplification was performed in a total reaction volume of 20 μL containing 10 ng of template DNA, 0.8 μL of each primer (10 μM), 4 μL of 5× FastPfu buffer, 0.4 μL of FastPfu polymerase, 0.2 μL of bovine serum albumin, and 250 μM dNTPs. The PCR program consisted of an initial denaturation at 95 °C for 3 min, followed by 29 cycles of denaturation at 95 °C for 30 s, annealing at 55 °C for 30 s, and extension at 72 °C for 45 s, with a final extension at 72 °C for 10 min. PCR products were examined by agarose gel electrophoresis, purified, quantified, and pooled in equimolar proportions before sequencing. Extraction blanks were included during DNA extraction, and PCR negative controls without template DNA were included during amplification to monitor potential contamination introduced during sample processing and PCR. No mock community was included in the present sequencing workflow.

### 2.4. Illumina Sequencing and Bioinformatic Processing

Purified amplicons were sequenced using an Illumina MiSeq PE300 platform (Illumina, San Diego, CA, USA) according to the standard protocols of Majorbio Bio-Pharm Technology Co., Ltd. (Shanghai, China). Raw sequence data were processed using the QIIME 2 pipeline (2026.7). Raw reads were demultiplexed and primer sequences were removed, followed by quality filtering, denoising, paired-end merging, and chimera removal using the DADA2 plugin (2026.7.0). Non-singleton amplicon sequence variants (ASVs) were retained for downstream analyses. Representative ASV sequences were aligned using MAFFT (v7), and a phylogenetic tree was constructed using FastTree2 (2.2.0). Taxonomic assignment was performed using the classify-sklearn naïve Bayes classifier against the SILVA Release 138 reference database. ASVs assigned to chloroplasts, mitochondria, eukaryotes, or other non-target lineages were removed from the feature table. The resulting filtered ASV abundance table was used for downstream community analyses.

### 2.5. Sequencing Coverage and Community Structure

Rarefaction curves based on the Chao1 richness index, Shannon diversity index, and Good’s coverage index were generated from the filtered ASV abundance table to evaluate sequencing sufficiency. Because no formal group-level alpha-diversity comparison was included in the present study, these indices were used to evaluate sequencing coverage and describe within-sample diversity patterns.

Beta diversity was assessed using Bray–Curtis dissimilarity calculated from ASV relative abundance profiles. Principal coordinate analysis (PCoA) was used to visualize differences in microbial community structure among EGC, MGC, and LGC. Overall differences among the three growth stages were evaluated using analysis of similarities (ANOSIM) with 999 permutations based on Bray–Curtis dissimilarity. Statistical significance was defined as *p* < 0.05. Taxonomic composition was summarized at the phylum, class, order, family, and genus levels based on relative abundance.

### 2.6. Shared and Stage-Specific Taxa

Venn diagrams were used to evaluate shared and stage-specific taxa among EGC, MGC, and LGC at the phylum, family, genus, and ASV levels. A taxon was considered present in a growth stage if it was detected in at least one of the five pooled intestinal samples from that stage. Taxa detected exclusively in one growth stage were considered stage-specific, whereas taxa detected in two or all three growth stages were assigned to the corresponding shared fractions.

### 2.7. Classification of Transient, Intermittent, and Persistent Taxa

To characterize cross-sample occurrence patterns across the complete dataset, the prevalence of each bacterial ASV was calculated across all 15 pooled intestinal samples as the proportion of samples in which that ASV was detected. Based on dataset-wide prevalence, ASVs were classified as transient (prevalence < 0.2), intermittent (0.2 ≤ prevalence ≤ 0.8), or persistent (prevalence > 0.8). Accordingly, persistent ASVs were detected in at least 13 of the 15 pooled samples.

The term “persistent” is used here strictly as an operational cross-sample prevalence category and does not imply longitudinal persistence of a microbial taxon within the same individual fish, because different fish were sampled at each time point. After dataset-wide classification, each sampling stage was evaluated separately. The number of detected ASVs belonging to the transient, intermittent, and persistent categories was expressed as a percentage of the total number of ASVs detected at each stage, and the cumulative relative abundance contributed by each prevalence category was calculated separately for EGC, MGC, and LGC.

### 2.8. Niche Breadth and Generalist–Specialist Classification

Niche breadth was calculated using Levins’ niche breadth index [27]:

B = 1/Σ(P_i_^2^)

where B represents the niche breadth of each ASV, and P_i_ represents the proportion of the total abundance of that ASV contributed by sample i. The calculation was based on abundance distributions across all 15 pooled intestinal samples. Zero-abundance values were retained as zero during calculation.

Niche-breadth analysis was performed using the built-in niche-breadth module of the Majorbio Cloud Platform (Majorbio Bio-Pharm Technology Co., Ltd., Shanghai, China) using the platform default parameters. ASVs with B < 1.5 were operationally classified as specialists, those with 1.5 ≤ B ≤ 2.0 as neutralists, and those with B > 2.0 as generalists. These fixed thresholds were applied to the complete dataset and were not estimated separately for individual growth stages. The numerical proportions of generalists, neutralists, and specialists were subsequently calculated across the dataset, and their cumulative relative abundance contributions were summarized separately for EGC, MGC, and LGC.

These categories describe the relative breadth of ASV abundance distributions within the present sample set and are therefore interpreted as statistical ecological classifications rather than direct measurements of the physiological niche breadth of individual bacterial taxa.

### 2.9. Statistical Analysis

Differences in the relative abundance of selected genera and ASVs among EGC, MGC, and LGC were evaluated using the Kruskal–Wallis H test. Because only five pooled samples were available per sampling stage and no multiple-testing correction was applied to these taxon-level comparisons, these analyses were treated as exploratory and hypothesis-generating. The displayed *p* values therefore represent nominal, unadjusted significance values and should not be interpreted as confirmatory evidence of differential abundance. Community-level differences were assessed using ANOSIM (2.7-6) as described above. Statistical significance was defined as *p* < 0.05.

### 2.10. Nucleotide Sequence Accession Number

The raw sequence data are available in the Genome Sequence Archive (Genomics, Proteomics & Bioinformatics 2021) at the National Genomics Data Center (Nucleic Acids Res 2022), China National Center for Bioinformation/Beijing Institute of Genomics, Chinese Academy of Sciences (accession codes: CRA049855).

### 2.11. Ethics Statement

All procedures involving *L. crocea* were reviewed and approved by the Science and Technology Ethics Review Committee of Ningde Normal University (Approval No. ND-010-2025; approved on 1 August 2025).

## 3. Results

### 3.1. Sequencing Coverage and Growth-Associated Differentiation of Gut Bacterial Communities

After sequence optimization and quality filtering, a total of 909,832 high-quality sequences were retained across the 15 pooled intestinal samples, with an average of 60,655 ± 20,006 sequences per sample and a range of 40,306–125,245 sequences per sample. Rarefaction curves based on Chao1 richness increased rapidly during the initial increase in sequencing depth and subsequently approached plateaus. Shannon diversity curves similarly stabilized with increasing read number, while Good’s coverage values approached 1.0 across all samples. These patterns indicated that sequencing depth was sufficient to capture the major detectable bacterial diversity in the 15 intestinal samples. Considerable variation in Chao1 and Shannon rarefaction profiles was visible both within and among growth stages. Several MGC samples showed relatively high estimated richness and diversity, while some LGC samples occupied the lower range of the Shannon profiles (Figure S1). Chao1 and Shannon indices were used here only to evaluate sequencing sufficiency and describe within-sample diversity patterns. Because no formal inferential comparison of alpha diversity was performed among EGC, MGC, and LGC, the present data do not support conclusions regarding statistically significant changes in alpha diversity across sampling stages.

**Figure 1:**
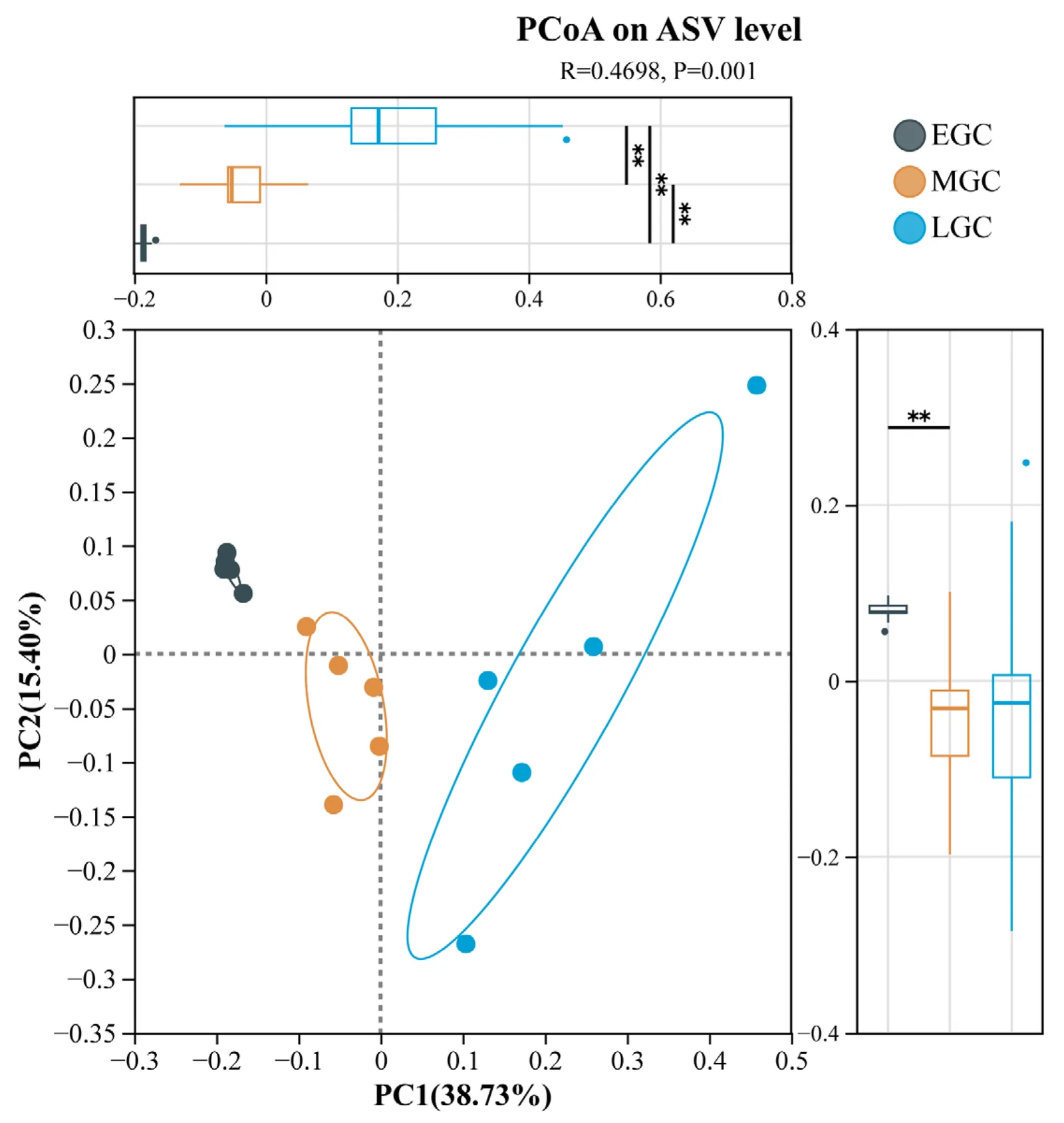
Principal coordinate analysis (PCoA) of gut bacterial community structure across three growth stages of *L. crocea*. PCoA was performed at the ASV level using Bray–Curtis dissimilarity calculated from relative abundance profiles. ** *p* < 0.01.

The ASV-level PCoA revealed clear differentiation in overall microbial community structure among growth stages (Figure 1). PC1 explained 38.73% of the total variation and PC2 explained 15.40%, together accounting for 54.13% of the variation in bacterial community composition. ANOSIM demonstrated a significant overall difference among EGC, MGC, and LGC (R = 0.4698, *p* = 0.001). EGC samples occupied a relatively compact region on the negative side of PC1, MGC samples were distributed nearer the central region, and LGC samples occurred predominantly on the positive side of PC1 with broader dispersion. ANOSIM based on 999 permutations indicated a significant overall difference among EGC, MGC, and LGC (R = 0.4698, *p* = 0.001). The moderate R value indicates partial rather than complete separation among the three sampling stages, consistent with the overlap visible in the PCoA ordination. Given the limited number of pooled samples per stage (n = 5), this result should be interpreted as evidence of stage-associated community differentiation within the present dataset rather than as strong separation among independently replicated experimental treatments.

### 3.2. A Conserved Phylum-Level Framework Masked Extensive Restructuring at Finer Taxonomic Levels

**Figure 2:**
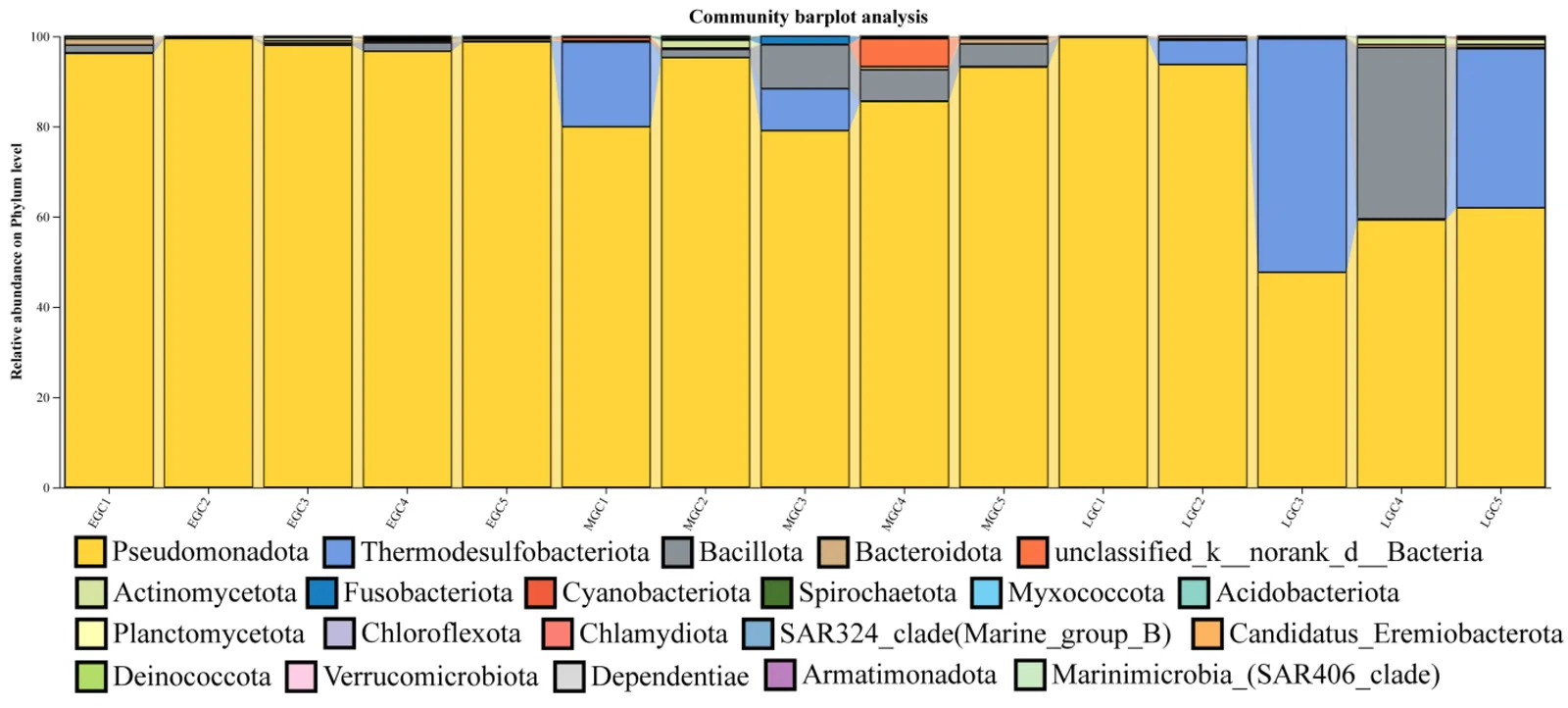
Relative abundance of gut bacterial taxa at the phylum level across three growth stages of *L. crocea*. Each bar represents one pooled intestinal sample.

*Pseudomonadota* represented the dominant bacterial phylum throughout all three growth stages. EGC samples showed particularly similar phylum-level profiles, with *Pseudomonadota* accounting for almost the entire community in most pools. Secondary phyla occurred only at relatively low abundance (Figure 2). MGC retained strong *Pseudomonadota* dominance but showed increased contributions from secondary phyla. *Thermodesulfobacteriota* became conspicuous in individual MGC samples, and *Bacillota* and several additional phyla also contributed greater proportions than in EGC. LGC displayed the most pronounced redistribution of major phyla. Some LGC pools remained overwhelmingly dominated by *Pseudomonadota*, whereas *Thermodesulfobacteriota* contributed approximately half of the community in one sample and *Bacillota* represented a major component in another. Consequently, later-stage compositional differentiation occurred largely through redistribution among secondary bacterial lineages rather than replacement of *Pseudomonadota* itself.

The same pattern became clearer at the class level. *Alphaproteobacteria* and *Gammaproteobacteria* dominated EGC and remained important across all stages. EGC generally showed high *Alphaproteobacteria* abundance together with a relatively consistent *Gammaproteobacteria* fraction. In MGC, the balance between these classes became more variable, while *Desulfovibrionia* and Clostridia increased in individual samples. LGC exhibited several distinct configurations in which *Gammaproteobacteria*, *Desulfovibrionia*, or Bacilli contributed strongly to individual pools (Figure S2).

At the order level, *Hyphomicrobiales* represented the principal EGC lineage and consistently accounted for a large proportion of the microbiota. *Burkholderiales* and *Pseudomonadales* represented other major components. In MGC, *Hyphomicrobiales* remained dominant but showed a lower and more variable contribution, accompanied by greater representation of *Desulfovibrionales*, *Enterobacterales*, *Lachnospirales*, and other orders. LGC showed substantially greater redistribution among dominant orders. *Burkholderiales* became particularly abundant in some samples, whereas *Desulfovibrionales* and *Mycoplasmatales* dominated substantial fractions of other LGC communities. These patterns indicate that later-stage microbial restructuring did not produce a single uniform replacement state (Figure S3).

Family-level profiles closely paralleled these changes. *Beijerinckiaceae* consistently dominated EGC, accompanied primarily by *Pseudomonadaceae*, *Moraxellaceae*, *Comamonadaceae*, and *Burkholderiaceae*. *Beijerinckiaceae* remained important in MGC but contributed a smaller proportion, while *Comamonadaceae* and several other families increased. LGC contained several substantially different dominant-family configurations. *Comamonadaceae* became highly abundant in individual pools, *Desulfovibrionaceae* dominated another, *Mycoplasmataceae* expanded strongly in one sample, and other LGC pools retained larger *Beijerinckiaceae* fractions (Figure S4).

### 3.3. Genus-Level Composition Revealed Strong Redistribution of Dominant Bacterial Members

**Figure 3:**
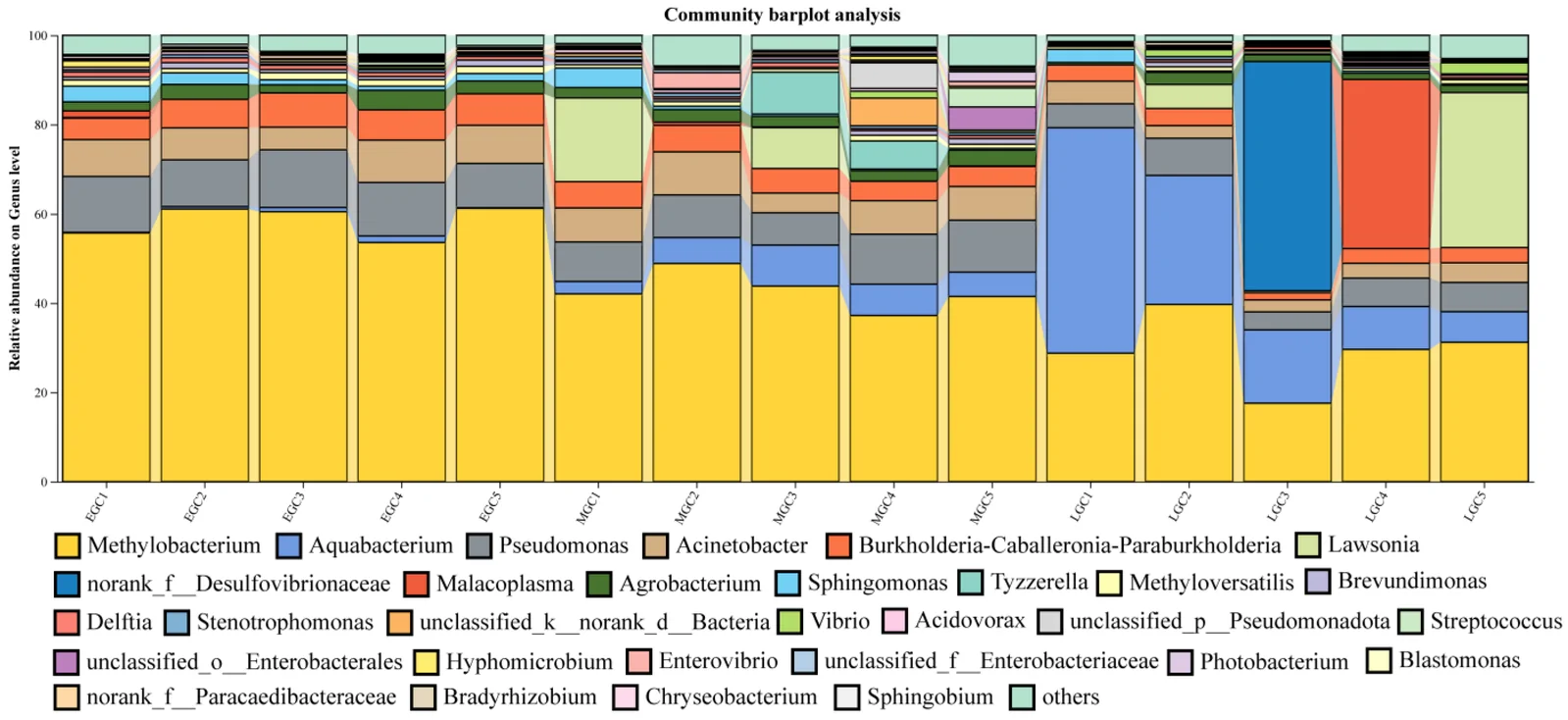
Relative abundance of dominant gut bacterial genera across three growth stages of *L. crocea*. Each bar represents one pooled intestinal sample.

Genus-level analysis revealed considerably stronger growth-associated differences than the phylum-level profiles. Dominant genera included *Methylobacterium*, *Aquabacterium*, *Pseudomonas*, *Acinetobacter*, *Burkholderia-Caballeronia-Paraburkholderia*, *Lawsonia*, *Malacoplasma*, *Agrobacterium*, *Sphingomonas*, *Tyzzerella*, *Methyloversatilis*, *Brevundimonas*, *Delftia*, and *Stenotrophomonas* (Figure 3).

EGC showed a comparatively reproducible genus-level composition. *Methylobacterium* consistently represented approximately half or more of the bacterial community in all five pools and therefore constituted the primary dominant genus at the smallest body-size class. *Pseudomonas* represented another major EGC member, while *Acinetobacter*, *Aquabacterium*, and *Burkholderia-Caballeronia-Paraburkholderia* contributed smaller but recurrent fractions. Most other genera remained at relatively low abundance. The MGC microbiota retained substantial *Methylobacterium* abundance but displayed a clear reduction in its dominance relative to EGC. *Aquabacterium* increased, and the relative contributions of *Pseudomonas*, *Acinetobacter*, *Burkholderia-Caballeronia-Paraburkholderia*, *Sphingomonas*, *Tyzzerella*, and other genera varied substantially among samples.

LGC exhibited the most extensive redistribution of dominant genera. *Methylobacterium* remained present but no longer accounted for the overwhelming fraction observed in EGC. Instead, different LGC pools exhibited strong enrichment of different bacterial lineages. *Aquabacterium* became a major component in several LGC samples. A *Desulfovibrionaceae*-affiliated taxon accounted for a very large fraction of another sample, *Malacoplasma* expanded strongly in one pool, and *Lawsonia* made a particularly large contribution in another.

Therefore, the transition from EGC to LGC was not characterized by the replacement of *Methylobacterium* by one single later-stage dominant taxon. Rather, its declining dominance was accompanied by expansion of multiple bacterial lineages, producing several distinct late-stage community configurations.

### 3.4. Stage-Specific Taxonomic Membership Increased at the Genus Level but Showed a Nonlinear ASV-Level Pattern

**Figure 4:**
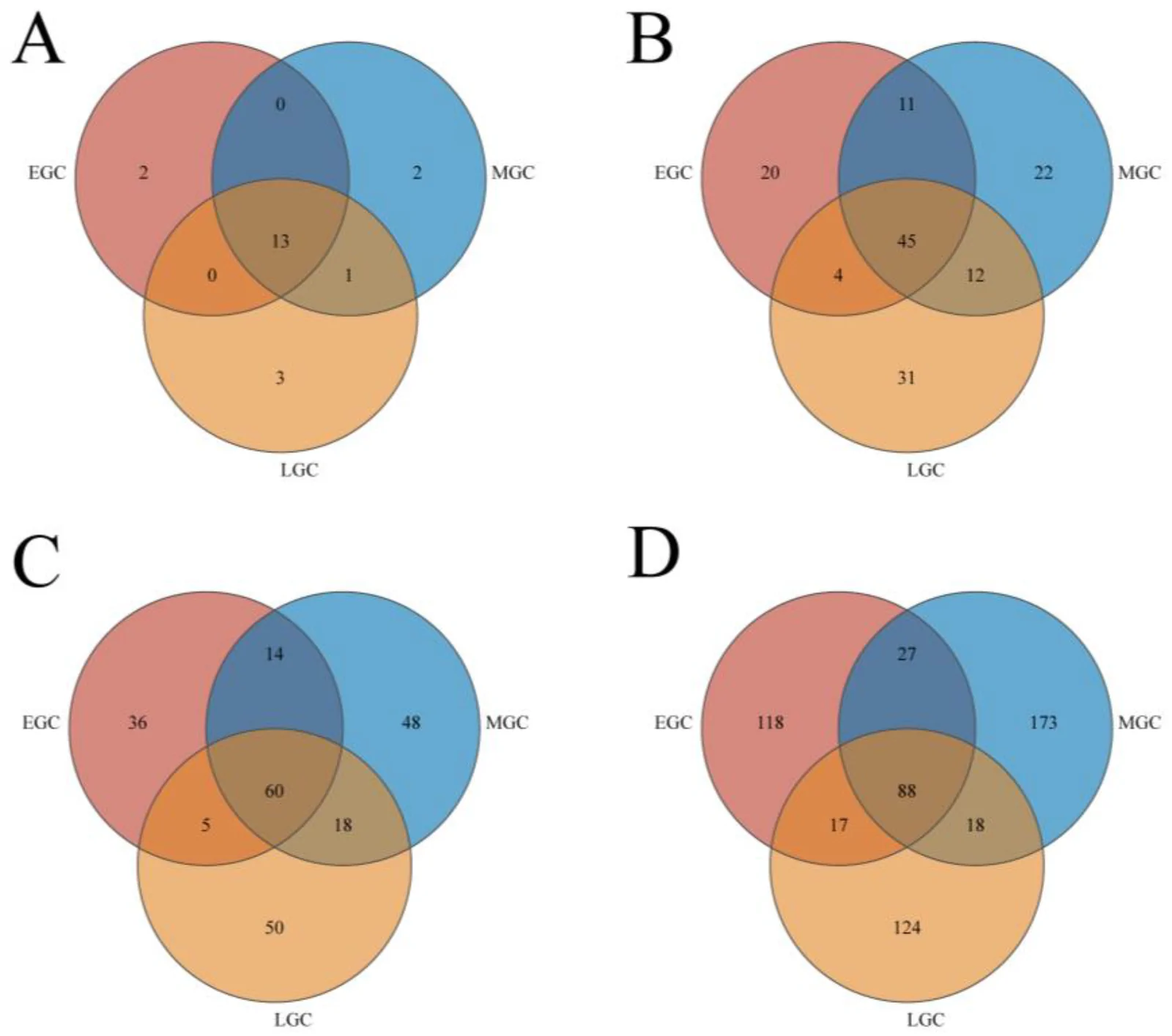
Shared and stage-specific bacterial taxa among the three growth stages of *L. crocea*. Venn diagrams show shared and unique taxa at the (**A**) phylum, (**B**) family, (**C**) genus, and (**D**) ASV levels among EGC, MGC, and LGC.

Venn analysis revealed increasing differentiation as taxonomic resolution increased (Figure 4). At the phylum level, 13 phyla were shared among all three growth stages. EGC and MGC each contained two unique phyla and LGC contained three. No phyla were shared exclusively between EGC and MGC or between EGC and LGC, while one phylum was shared exclusively between MGC and LGC. At the family level, 45 families were shared among all three groups. EGC contained 20 unique families, MGC contained 22, and LGC contained 31. Eleven additional families were shared exclusively between EGC and MGC, four between EGC and LGC, and 12 between MGC and LGC. The growth-associated increase in stage specificity became more evident at the genus level. Sixty genera were shared across all three stages, while the number of unique genera increased from 36 in EGC to 48 in MGC and 50 in LGC. Fourteen genera occurred only in EGC and MGC, five only in EGC and LGC, and 18 only in MGC and LGC. Thus, later-stage communities did not undergo a contraction of genus-level membership. Instead, LGC contained the largest number of stage-specific genera.

The ASV-level pattern was more complex. Eighty-eight ASVs were shared among EGC, MGC, and LGC. EGC contained 118 unique ASVs, MGC contained 173, and LGC contained 124. Twenty-seven additional ASVs were shared between EGC and MGC, 17 between EGC and LGC, and 18 between MGC and LGC. MGC therefore contained the largest stage-specific ASV pool, whereas LGC contained the largest numbers of stage-specific genera and families. These descriptive patterns indicate that stage-specific membership did not follow a simple monotonic trajectory across taxonomic resolutions. Because no inferential test was applied to the aggregate numbers of stage-specific taxa in the Venn analysis, these differences are interpreted descriptively rather than as statistically significant stage-level changes.

### 3.5. Persistent Taxa Maintained Their Numerical Representation but Progressively Lost Abundance Dominance

**Figure 5:**
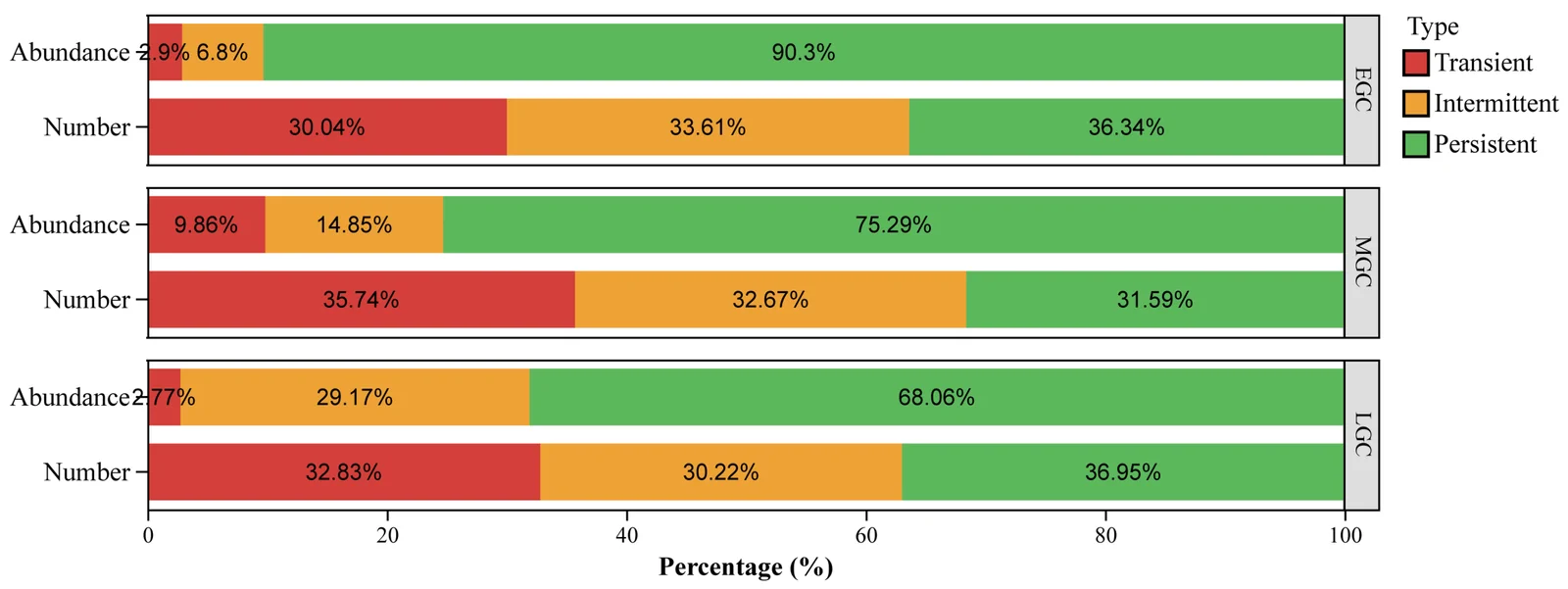
Relative representation and abundance contribution of transient, intermittent, and persistent ASVs across three growth stages of *L. crocea*. ASV prevalence was calculated across all 15 pooled intestinal samples. ASVs were classified as transient (prevalence < 0.2), intermittent (0.2 ≤ prevalence ≤ 0.8), or persistent (prevalence > 0.8). “Number” represents the percentage of detected ASVs assigned to each prevalence category, whereas “Abundance” represents their cumulative relative abundance contribution within each growth stage.

All percentage values reported in the persistence analysis represent proportional contributions calculated from sequencing-derived relative abundance data and should not be interpreted as absolute microbial biomass, cell abundance, or bacterial load. Occurrence-based classification revealed a marked difference between the numerical representation and abundance contribution of persistent taxa (Figure 5). In EGC, transient taxa represented 30.04% of detected taxa, intermittent taxa represented 33.61%, and persistent taxa accounted for 36.34%. Thus, the three categories were relatively balanced in taxon number. Their abundance contributions, however, were highly unequal. Persistent taxa contributed 90.30% of the total bacterial community, whereas intermittent taxa accounted for only 6.80% and transient taxa for 2.90%. The smallest fish therefore harbored numerous intermittent and transient taxa by number, but community abundance was overwhelmingly concentrated within persistent members.

In MGC, transient taxa accounted for 35.74% of detected taxa, intermittent taxa for 32.67%, and persistent taxa for 31.59%. Persistent taxa therefore remained numerically comparable to the other categories rather than disappearing from the community. Their abundance dominance nevertheless declined substantially to 75.29%. Meanwhile, intermittent taxa increased to 14.85% of the total abundance and transient taxa increased temporarily to 9.86%. The LGC pattern further emphasized the distinction between persistence and abundance. Persistent taxa represented 36.95% of detected taxa—slightly higher than their numerical proportion in EGC—whereas transient and intermittent taxa represented 32.83% and 30.22%, respectively.

Despite their maintained numerical representation, persistent taxa contributed only 68.06% of the total abundance in LGC. In contrast, intermittent taxa increased dramatically to 29.17%, while transient taxa contributed 2.77%. Thus, the major developmental change was not the numerical replacement of persistent taxa. Instead, persistent taxa progressively lost quantitative dominance as intermittently occurring taxa became increasingly important components of community abundance.

### 3.6. Specialists Were Numerically Dominant and Increased Their Contribution to Later-Stage Communities

**Figure 6:**
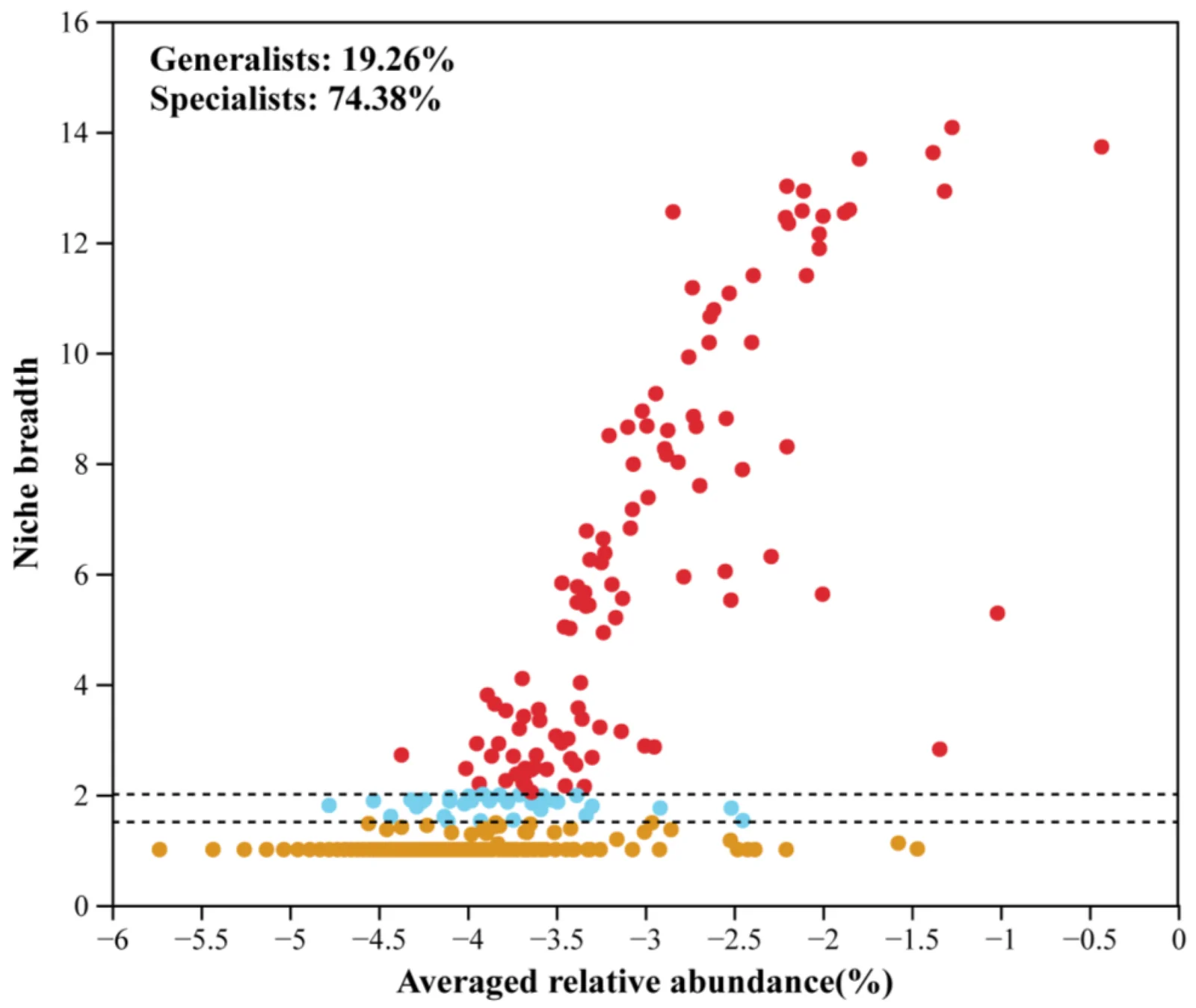
Niche-breadth distribution of gut bacterial ASVs in *L. crocea*. Each point represents one ASV. Dashed horizontal lines indicate the classification thresholds at B = 1.5 and B = 2.0 used to distinguish specialists, neutralists, and generalists. Red dots represent generalists, blue dots represent neutralists, and yellow dots represent specialists.

Likewise, the abundance contributions of generalists, neutralists, and specialists represent relative sequence-abundance proportions rather than absolute microbial biomass or cell counts. Niche-breadth analysis identified distinct ecological strategies among gut microbial taxa (Figure 6). Across the dataset, 74.38% of classified taxa were identified as specialists, whereas only 19.26% were classified as generalists; the remaining fraction represented neutralists. Specialists were therefore substantially more numerous than generalists. The niche-breadth distribution showed that specialists clustered within the narrowest niche-width range, whereas generalists occupied much broader niche widths and frequently occurred at higher average relative abundances.

**Figure 7:**
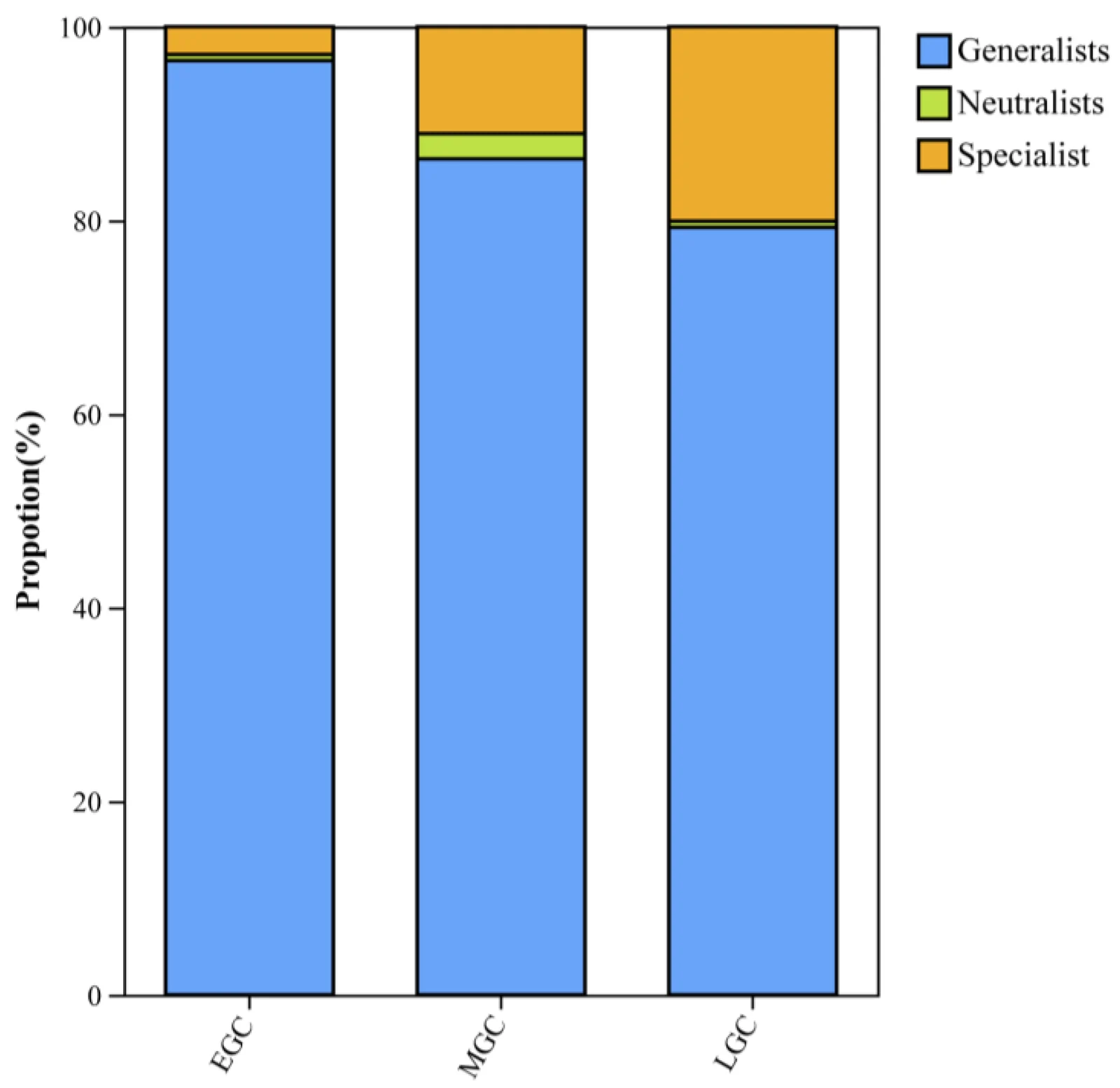
Relative abundance contributions of microbial generalists, neutralists, and specialists across EGC, MGC, and LGC.

The numerical predominance of specialists contrasted strongly with their community-level abundance contributions (Figure 7). EGC was overwhelmingly dominated by generalists, which contributed approximately 95% of the bacterial abundance, whereas specialists made only a minor contribution. MGC retained generalist dominance, but their proportional contribution decreased and specialists increased. This shift became most evident in LGC. Generalists still accounted for the majority of total abundance but declined to approximately 80%, while specialist contribution increased to close to one-fifth of the bacterial community.

The niche-breadth results therefore revealed a clear growth-associated redistribution: specialists were abundant in taxon number throughout the dataset but became progressively more important in community abundance as fish grew. This trajectory paralleled the decline in persistent-taxon dominance and increase in intermittent taxa. However, because persistence and niche breadth were independently classified, these parallel trends do not demonstrate that persistent taxa were generalists or that intermittent taxa were specialists.

### 3.7. Dominant Genera Exhibited Contrasting Growth-Associated Trajectories

**Figure 8:**
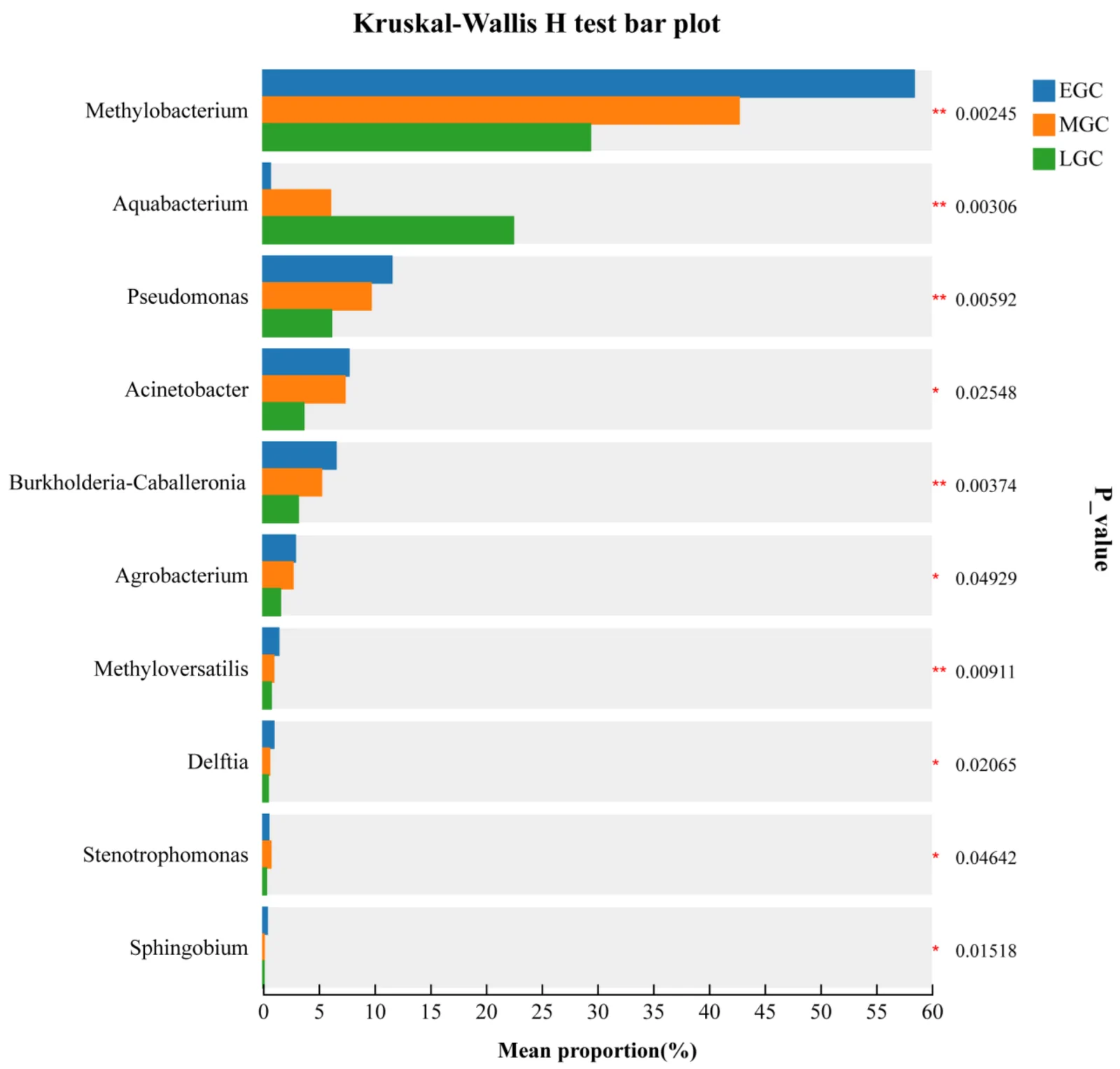
Candidate bacterial genera showing exploratory growth-stage-associated differences in the gut microbiota of *L. crocea*. Differences were evaluated using the Kruskal–Wallis H test. Displayed *p* values are nominal and were not corrected for multiple comparisons; * *p* < 0.05 and ** *p* < 0.01.

Kruskal–Wallis analysis identified ten genera displaying nominal differences among growth stages: *Methylobacterium*, *Aquabacterium*, *Pseudomonas*, *Acinetobacter*, *Burkholderia-Caballeronia*, *Agrobacterium*, *Methyloversatilis*, *Delftia*, *Stenotrophomonas*, and *Sphingobium* (Figure 8).

*Methylobacterium* showed the clearest decreasing trajectory. Its mean relative abundance was highest in EGC, declined substantially in MGC, and reached its lowest value in LGC. *Aquabacterium* exhibited an opposing pattern. It represented only a minor component in EGC, increased markedly in MGC, and reached its highest mean abundance in LGC. *Pseudomonas* also declined progressively from EGC to LGC. Similar overall decreases were observed for *Acinetobacter* and *Burkholderia-Caballeronia*. *Agrobacterium*, *Methyloversatilis*, *Delftia*, and *Sphingobium* likewise showed higher proportions in early-stage fish, although their total community contributions were smaller. *Stenotrophomonas* exhibited a more complex stage-dependent pattern. The genus-level results therefore demonstrate that community restructuring involved coordinated changes among multiple dominant bacterial lineages, rather than a single taxonomic replacement event.

ASV-level analysis identified ten sequence variants showing nominal growth-stage-associated differences, with *p* values ranging from 0.00245 to 0.04929 (Figure S5). ASV2 represented the most abundant differential ASV and showed a pronounced decrease from EGC through MGC to LGC. ASV3, ASV6, and ASV4 exhibited similar decreasing trajectories, although their overall abundance was lower. ASV10, ASV16, ASV23, and ASV34 likewise contributed more strongly to EGC and declined in later stages. In contrast, ASV1 exhibited the opposite pattern, showing very low abundance in EGC, an intermediate abundance in MGC, and a marked increase in LGC. ASV8 differed from both patterns by showing its highest abundance in MGC, indicating a stage-specific response rather than a monotonic trajectory. These fine-scale results further show that growth-associated succession included both monotonic changes and MGC-specific enrichment among individual ASVs.

## 4. Discussion

### 4.1. Growth Was Associated with Pronounced but Nonlinear Gut Microbial Succession

The significant differentiation among EGC, MGC, and LGC was consistent with pronounced growth-associated restructuring of the *L. crocea* gut microbiota. Ontogenetic microbial restructuring has been widely reported in fishes, including *Danio rerio*, *Ictalurus punctatus* and *Salmo salar* [6,7,8]. These studies further show that developmental microbiome trajectories can be nonlinear and may contain distinct transitional community states rather than simply moving from an immature to a uniformly stable mature microbiota. This interpretation is particularly relevant to *L. crocea*. Wei reported strong age-associated changes from larval stages to one-year-old fish, without a simple uniform developmental trend [16]. More recently, Li identified stage-specific microbial transitions during early digestive-system development [17]. Thus, the strong differentiation observed here across 5–8 g, 39–46 g, and 160–162 g fish extends previous evidence of microbiome succession into later juvenile/grow-out size classes. Because the three groups received feed with the same formulation, categorical changes in diet composition are unlikely to explain the observed succession. However, feeding quantity increased with host size, while culture duration and developmental status necessarily covaried with body weight. Therefore, the present experiment cannot isolate body mass as an independent causal variable.

### 4.2. Stable Phylum-Level Dominance Did Not Imply Microbiome Stability

*Pseudomonadota* remained dominant throughout all three growth stages, but this apparent high-level stability masked extensive reorganization at finer taxonomic levels. Similar patterns have been reported across fish microbiome systems, in which broad phylum-level composition remains relatively conserved while individual bacterial populations vary strongly according to host age, diet, environment, and intestinal niche [3,4,5]. The present data provide a particularly clear example. EGC was consistently dominated by *Methylobacterium*, whereas later-stage communities showed increased contributions from *Aquabacterium*, *Desulfovibrionaceae*-associated taxa, *Malacoplasma*, *Lawsonia*, and other lineages. This distinction is relevant to previous *L. crocea* studies, because diet, probiotics, rearing conditions, and disease status have all been shown to produce substantial changes among intestinal bacterial taxa without necessarily producing complete replacement of dominant phyla [19,20,21,22,23,24,25,26,27].

### 4.3. MGC Represents a Distinct Transitional Phase of Microbial Turnover

MGC contained the highest number of stage-specific ASVs, distinguishing the intermediate size class from both EGC and LGC. If microbiome succession simply involved progressive accumulation of taxa, LGC would be expected to contain the greatest unique ASV pool. Conversely, if development involved progressive taxonomic contraction, EGC would contain the largest pool. Neither pattern occurred. Instead, MGC contained 173 unique ASVs, compared with 118 in EGC and 124 in LGC. This result indicates especially intense fine-scale turnover at the intermediate stage. The persistence data support the same interpretation. Persistent-taxon abundance declined sharply between EGC and MGC, while transient taxa temporarily increased to almost 10%. By LGC, the transient fraction declined again, whereas intermittent taxa became quantitatively much more important.

Stage-specific transitional communities have also been observed in *Danio rerio* and *Salmo salar* [6,9], and recent *L. crocea* developmental research identified discrete microbiome transitions during early digestive development [17]. Because the present groups were defined by body weight rather than histologically identified developmental stages, MGC represents a transitional microbiome configuration in the present dataset, whereas its correspondence to a defined physiological maturation stage remains unresolved.

### 4.4. Persistent Taxa Remained Present but Lost Their Strong Quantitative Dominance

The prevalence-based persistence analysis provided an additional perspective on community organization across the successive sampling stages. Here, “persistence” refers to repeated detection across pooled samples within the complete dataset rather than longitudinal persistence within individual fish. Thus, the analysis identifies microbial members that were broadly recurrent across the sampled population and sampling stages but does not demonstrate continuous colonization of individual hosts through time. Their abundance contribution, however, declined by more than 22 percentage points, from 90.30% in EGC to 68.06% in LGC. Thus, the key developmental change was loss of abundance dominance rather than loss of persistence. This distinction is important because persistence and abundance describe different ecological dimensions. Core-microbiome research in *Danio rerio* and other fishes has demonstrated that certain microorganisms can persist across individuals, treatments, environments, or time [12,13]. Persistence does not imply constant abundance, and a recurrent member may remain detectable while its proportional dominance changes considerably.

In EGC, relatively few persistent members collectively accounted for almost the entire microbial community. Such strong abundance concentration indicates that the early-stage community structure was dominated by a recurrent bacterial fraction. As fish grew, intermittent taxa contributed an increasingly large proportion of the microbiota, reaching 29.17% in LGC. Importantly, this increase occurred without a corresponding major increase in the numerical proportion of intermittent taxa. Consequently, later-stage restructuring primarily reflected changes in abundance allocation among occupancy classes rather than wholesale replacement of persistent membership. This differs markedly from developmental systems in which recurrent/core microorganisms become increasingly dominant with age, such as patterns reported during early development of *Trichinotus Blochii* [11]. The present *L. crocea* trajectory therefore differs from a simple developmental pattern in which persistent taxa become progressively more dominant with age.

### 4.5. Increasing Specialist Contributions Reveal Growth-Associated Changes in Microbial Niche Structure

Specialists represented nearly three-quarters of the classified ASVs according to the operational Levins niche-breadth thresholds used in this study, indicating that most taxa showed relatively restricted abundance distributions across the 15 pooled samples. This statistical classification should not be interpreted as direct evidence that these microorganisms possess intrinsically narrow physiological or host niches. However, EGC community abundance was overwhelmingly dominated by generalists. Thus, numerical prevalence and quantitative importance were strongly decoupled. As host size increased, generalist contribution declined while specialist contribution increased. Specialists therefore became progressively more important to the quantitative structure of MGC and especially LGC. Ecologically, generalists and specialists are expected to differ in their ability to occupy heterogeneous environments, respond to changing conditions, and contribute to community organization [14,15]. Large-scale microbial analyses have shown that broad-niche taxa and narrow-niche taxa can exhibit contrasting ecological and evolutionary characteristics [14,15]. In a developing intestinal system, increasing contribution from specialists could reflect greater differentiation of available microbial niches or increased importance of taxa adapted to more specific host-associated conditions.

The present study did not measure intestinal physicochemical gradients or experimentally manipulate microbial niches; therefore, the increased specialist contribution indicates greater quantitative representation of narrow-niche taxa but does not demonstrate increased intestinal niche complexity. Persistent-taxon dominance decreased in parallel with increasing specialist contribution. Because persistence and niche breadth were classified independently, these concurrent trends do not establish that later-stage communities were enriched specifically in taxa that were both less prevalent and narrower in niche breadth.

### 4.6. Opposing Trajectories of Dominant Taxa Illustrate Fine-Scale Community Replacement

The differential-abundance results provide taxonomic examples of the broader ecological restructuring. *Methylobacterium* declined strongly across the three stages. Its high abundance and consistency in EGC indicate that it contributed substantially to the strong early-stage dominance structure. In contrast, *Aquabacterium* increased markedly and reached its greatest contribution in LGC. Several other common genera, including *Pseudomonas*, *Acinetobacter*, and *Burkholderia-Caballeronia*, generally declined. The ASV results similarly identified both decreasing and increasing sequence variants, as well as MGC-specific responses. These patterns demonstrate that gut succession involved multiple simultaneous trajectories rather than uniform directional change across all microorganisms.

These taxonomic patterns do not establish microbial function. The ecological roles of genera cannot be inferred directly from 16S rRNA gene abundance, and individual species or strains within the same genus may differ substantially in metabolic activity and host interactions [2]. Previous *L. crocea* studies have experimentally linked dietary or probiotic treatments to changes in intestinal microbial composition alongside host physiological responses [18,19,20,21,22], but the present study did not measure microbial gene expression, metabolites, or isolate-specific functions. Accordingly, *Methylobacterium*, *Aquabacterium*, and the other differential taxa are interpreted here as candidate indicators of growth-associated microbiome turnover rather than as functionally defined beneficial or detrimental bacteria.

### 4.7. Study Design Considerations and Limitations

Several aspects of the experimental design should be considered when interpreting the present results. First, the three body-size classes were sampled successively from the same cultured population maintained in a single pond. Consequently, body size, host age, culture duration, and chronological sampling time covaried and could not be separated experimentally. The observed microbial differences therefore represent temporal patterns associated with host growth under the conditions of this culture sequence, rather than effects that can be attributed exclusively to increasing body size or host development. Second, the five pooled intestinal samples obtained at each sampling stage represented bio-logical subsamples from fish within the same pond rather than independent pond-level replicates. Accordingly, statistical differences among EGC, MGC, and LGC describe with-in-pond temporal differentiation and should not be extrapolated as replicated treatment effects at the pond level. The relatively small number of pooled samples per sampling stage (n = 5) limited statistical power. Accordingly, taxon-level Kruskal–Wallis comparisons were considered exploratory, and nominal *p* values were not corrected for multiple testing. These candidate growth-stage-associated taxa therefore require validation in independent datasets with greater replication and multiple-testing-adjusted statistical frameworks. Third, concurrent measurements of water temperature, salinity, dissolved oxygen, pH, ammonia, nitrite, and environmental microbial communities were not available. Temporal changes in these environmental factors may therefore have contributed to the observed microbiome differences. Although feed formulation remained unchanged throughout cultivation, feeding quantity increased with fish size and represents an additional covarying factor. Finally, pooling intestinal tissues from three fish into each analytical sample reduced the influence of extreme individual profiles but prevented direct quantification of inter-individual microbiome variability. Therefore, the present study characterizes pooled population-level patterns within each sampling stage rather than microbiome heterogeneity among individual fish. Future studies incorporating replicated culture units, simultaneous environmental monitoring, and host physiological measurements will be necessary to disentangle the relative contributions of host development and temporal environmental variation to gut microbiome succession in *L. crocea*.

## 5. Conclusions

The gut microbiota of *L. crocea* showed pronounced ecological restructuring across three successive size- and age-associated sampling stages. Although *Pseudomonadota* remained the dominant phylum, substantial turnover occurred at finer taxonomic resolutions, including a progressive reduction in *Methylobacterium* dominance and increased representation of several alternative bacterial lineages. Microbial membership changed nonlinearly, with stage-specific genera increasing toward the later sampling stage whereas unique ASVs peaked in MGC, indicating particularly pronounced fine-scale turnover at the intermediate sampling point. However, because growth stages were defined according to body size and culture duration rather than histologically verified developmental or physiological maturation criteria, MGC should not be interpreted as a formally defined physiological transitional stage. Persistent ASVs remained numerically well represented across the dataset, but their contribution to total community abundance decreased from 90.30% in EGC to 68.06% in LGC, whereas intermittent ASVs increased from 6.80% to 29.17%. In parallel, generalists remained quantitatively dominant, whereas the contribution of operationally classified specialists increased in later-stage communities. Together, these results demonstrate a substantial redistribution of microbial dominance, prevalence patterns, and inferred niche strategies across successive sampling stages of cultured *L. crocea*. Because host age and body size covaried with chronological sampling time within a single pond, these patterns should be interpreted as growth-associated temporal micro-biome restructuring rather than as evidence for effects caused exclusively by host growth. Microbial prevalence and niche breadth nevertheless provide complementary ecological dimensions for describing gut microbiome variation during the post-juvenile culture period of *L. crocea*. Because body size, host age, culture duration, feeding quantity, and chronological sampling time covaried in the present single-pond design, the observed microbiome restructuring cannot be causally attributed to body mass per se.

## Data Availability

The raw sequence data are available in the Genome Sequence Archive (Genomics, Proteomics & Bioinformatics 2021) at the National Genomics Data Center (Nucleic Acids Res 2022), China National Center for Bioinformation/Beijing Institute of Genomics, Chinese Academy of Sciences (accession codes: CRA049855).

## Supplementary Materials

The following supporting information can be downloaded at: https://www.mdpi.com/article/10.3390/biology15201807/s1, Figure S1. Rarefaction curves of gut bacterial communities across three growth stages of *L. crocea*. Panels show (**A**) Chao1 richness, (**B**) Shannon diversity, and (**C**) Good’s coverage. Each curve represents one pooled intestinal sample. Figure S2. Relative abundance of gut bacterial taxa at the class level across EGC, MGC, and LGC. Figure S3. Relative abundance of gut bacterial taxa at the order level across EGC, MGC, and LGC. Figure S4. Relative abundance of gut bacterial taxa at the family level across EGC, MGC, and LGC. Figure S5. Candidate ASVs showing nominal growth-stage-associated differences in the gut microbiota of *L. crocea*. Bars show mean relative abundance in EGC, MGC, and LGC. Differences among growth stages were evaluated using the Kruskal–Wallis H test. Displayed *p* values are nominal and were not corrected for multiple comparisons; * *p* < 0.05 and ** *p* < 0.01.
